# The microbiome, resistome, and their co-evolution in sewage at a hospital for infectious diseases in Shanghai, China

**DOI:** 10.1128/spectrum.03900-23

**Published:** 2023-12-22

**Authors:** Yingying Ma, Nannan Wu, Tao Zhang, Yanpeng Li, Le Cao, Peng Zhang, Zhigang Zhang, Tongyu Zhu, Chiyu Zhang

**Affiliations:** 1 Shanghai Public Health Clinical Center, Fudan University, Shanghai, China; 2 State Key Laboratory for Conservation and Utilization of Bio-Resources in Yunnan, School of Life Sciences, Yunnan University, Kunming, Yunnan, China; 3 Shanghai Key Laboratory of Organ Transplantation, Zhongshan Hospital, Fudan University, Shanghai, China; University of Guelph College of Biological Science, Guelph, Ontario, Canada

**Keywords:** hospital, sewage, antibiotic resistance gene, antibiotic resistance bacteria, mobile genetic elements, metagenomic

## Abstract

The emergence of antibiotic-resistant bacteria (ARB) caused by the overuse of antibiotics severely threatens human health. Hospital sewage may be a key transmission hub for ARB. However, the complex link between the microbiome and resistomeresistance in hospital sewage remains unclear. In this study, metagenomic assembly and binning methods were used to investigate the microbial community, resistome, and association of antibiotic resistance genes (ARGs) with ARB in sewage from 10 representative sites (outpatient building, surgery building, internal medicine buildings [IMB1-4], staff dormitory, laboratory animal building, tuberculosis building [TBB], and hospital wastewater treatment plant) of a hospital in Shanghai from June 2021 to February 2022. A total of 252 ARG subtypes, belonging to 17 antibiotic classes, were identified. The relative abundance of KPC-2 was higher at IMBs and TBB than at other sites. Of the ARG-carrying contigs, 47.3%-62.6% were associated with mobile genetic elements, and the proportion of plasmid-associated ARGs was significantly higher than that of chromosome-associated ARGs. Although a similar microbiome composition was shared, certain bacteria were enriched at different sites. Potential pathogens *Enterococcus B faecium* and *Klebsiella pneumoniae* were primarily enriched in IMB2 and IMB4, respectively. The same ARGs were identified in diverse bacterial hosts (especially pathogenic bacteria), and accordingly, the latter possessed multiple ARGs. Furthermore, gene flow was frequently observed in the sewage of different buildings. The results provide crucial information on the characterization profiles of resistomes in hospital sewage in Shanghai.

**IMPORTANCE:**

Environmental antibiotic resistance genes (ARGs) play a critical role in the emergence and spread of antimicrobial resistance, which poses a global health threat. Wastewater from healthcare facilities serves as a significant reservoir for ARGs. Here, we characterized the microbial community along with the resistome (comprising all antibiotic resistance genes) in wastewater from a specialized hospital for infectious diseases in Shanghai. Potential pathogenic bacteria (e.g., *Escherichia coli*, *Pseudomonas aeruginosa*, *Klebsiella pneumoniae*, *Enterococcus B faecium*) were frequently detected in hospital wastewater and carried multiple ARGs. A complex link between microbiome and resistome was observed in the wastewater of this hospital. The monitoring of ARGs and antibiotic-resistant bacteria (ARB) in hospital wastewater might be of great significance for preventing the spread of ARB.

## OBSERVATION

The overuse and misuse of antibiotics in healthcare and agriculture have led to the increasing emergence and spread of antimicrobial resistance (AMR), which has become a serious threat to public health worldwide. It was estimated that drug-resistant infections cause at least 700,000 deaths each year, and the number will reach 10 million by 2050 if the problem of AMR is not addressed (1, 2). AMR-related death has become the second leading cause of death globally (3). Six pathogens, including *Escherichia coli*, *Staphylococcus aureus*, *Klebsiella pneumoniae*, *Streptococcus pneumoniae*, *Acinetobacter baumannii*, and *Pseudomonas aeruginosa*, contributed to the major burden of AMR and were listed as priority pathogens by the World Health Organization (4). The Global Antimicrobial Resistance and Use Surveillance System reported that about 42% of bloodstream infections were caused by third-generation cephalosporin-resistant *E. coli* (5). During 2011–2014, an increased prevalence of *Klebsiella pneumoniae* resistant to fluoroquinolones, third-generation cephalosporin, and/or aminoglycosides was observed in Europe (6). AMR rates vary largely across different countries and regions, and low-income countries have a higher AMR rate than developed countries (5, 6).

Antibiotic-resistant bacteria (ARB) and antibiotic resistance genes (ARGs) are not only present in humans and animals but also widely distributed in environments, including water bodies and soil (7–10). Recent evidence showed that the hospital environment harbors opportunistic ARB that often cause hospital-acquired infections (HAIs) and increase morbidity and mortality (7). The hospital environment is a complex system for the emergence and spread of ARB. Hospital sewage, which contains a high concentration of antibiotics, various bacteria, and ARB from the urine and feces of patients as well as medical waste, may serve as a key transmission hub for ARB and ARGs. The presence of high concentrations of various antibiotics in sewage imposes a strong selective pressure on the survival of ARB by increasing mutation rates and gene transfer frequency (11). The frequent occurrence of horizontal gene transfers mediated by mobile genetic elements (MGEs), including plasmids, and integrative and conjugative elements (ICEs), presents a public health concern for the generation of multidrug-resistant organisms (8). Furthermore, a partial microbial community survived in post-chlorinated effluents (11), suggesting that the chlorination disinfection process partially eliminated the bacteria in hospital sewage. Therefore, sewage is believed to be an important reservoir for the generation and spread of novel ARBs and ARGs (12).

China is the largest producer and consumer of antibiotics in the world. Half of all prescriptions in hospitals in China are involved in antibiotics (13), and its average daily antibiotic use (per 1,000 residents) was more than five times higher than those in the USA and UK (14). The overuse of antibiotics led to a nation-wide high prevalence of AMR. For example, carbapenem-resistant *Klebsiella pneumoniae* increased from 3% to 21% during 2005–2017 (15). In 2020, the national average rates of carbapenem-resistant *Escherichia coli*, carbapenem-resistant *Pseudomonas aeruginosa*, and carbapenem-resistant *Acinetobacter baumannii* were reported to be 1.6%, 18.3%, and 53.7%, respectively, and the rates of third-generation cephalosporin-resistant *Escherichia coli* and *Klebsiella pneumoniae* were 51.6% and 31.1%, respectively (16).

Characterizing microbiome and resistome profiles in hospital sewage is important for the surveillance of ARB and ARGs and the prevention of potential HAIs. Shanghai Public Health Clinical Center (SPHCC) is the unique 3A-grade specialized hospital for major infectious diseases (e.g., severe acute respiratory syndrome coronavirus 2 [SARS-CoV-2], HIV-1, hepatitis C virus [HCV], hepatitis B virus [HBV], and tuberculosis [TB]) in Shanghai. Here, we conducted a near-one-year study on the microbiome, resistome, and virulome in sewage collected at 10 SPHCC buildings from June 2021 to February 2022. The results not only provide crucial information for the prevention of HAIs and the elimination of ARB and ARGs but also partly present the state of ARB and ARG distribution in sewage in Shanghai.

### Sample collection

Sewage samples were collected from 10 SPHCC sites at a 2-month interval from June 2021 to February 2022. The sites include internal medicine buildings (IMB1, liver diseases; IMB2, departments of infectious diseases, neurology department, and respiratory medicine; IMB3, AIDS center; and IMB4, department of gastroenterology), surgery building (SB), outpatient building (OB), laboratory animal building (LAB), hospital wastewater treatment plant (HWTP), staff dormitory (SD), and tuberculosis building (TBB) (Fig. S1a). Sewage samples of 500 mL each were collected from 10 sites at five time points (IMB1, 4 samples; IMB2, 5 samples; IMB3, 5 samples; IMB4, 5 samples; SB, 5 samples; OB, 5 samples; LAB, 5 samples; HWTP, 3 samples; SD, 5 samples; TBB, 4 samples) (Fig. S1b). The sample number was not the same for each site because two sites were closed off due to receiving coronavirus disease 2019 (COVID-19) patients since December 2021 (Fig. S1b). A total of 46 samples were collected in sterile bottles and immediately stored at 4°C. Sample collections were taken on a day without rainfall.

### DNA extraction and metagenomic sequencing

Each sample was centrifuged at 10,000 × *g* for 30 min at 4°C on the second day of sampling, and the pellet was resuspended in sodium phosphate buffer. DNA was extracted using the FastDNA Spin Kit for Soil (MP Biomedicals, France). The DNA library was prepared using the Nextera XT DNA library preparation kit (Illumina, USA) and sequenced by the Illumina Novaseq platform with 2× 150 bp paired reads.

### Bacterial taxonomic profiling in hospital sewage

After removing four samples with low-quality DNA, 42 samples were used for the metagenomics sequencing (Fig. S1b). The low-quality reads were removed, and the sequencing adaptor was trimmed by Trimmomatic v.0.39 (17). The human reads were removed by mapping to the hg37 reference using Botiwe2 v.2.2.5 (18). The microbial reads were profiled using MetaPhlAn4 v.4.0.6 (19).

Beta diversity of bacterial taxonomic profiles was calculated based on Bray-Curtis distance using the vegan R package, and alpha diversity (observed species and evenness) was calculated using the R package. Lefse analysis was performed to identify discriminative microbiota features at different sites. The linear discriminant analysis (LDA) was used to analyze specific taxa at different sites based on the relative abundance of each taxon. A logarithmic LDA score >2 was used to determine discriminative features.

### Metagenomic assembly and identification of ARG-carrying contigs

The contigs of each sample were assembled by metaSPAdes v.3.13.0 with -min-contig-len 1,000 (20). Open reading frames (ORFs) were predicted using Prodigal v.2.6.3 (21). The ARG-carrying contigs (ACCs) and virulence factors (VFs) carrying contigs were identified using BLASTX against the Structured ARG database v.2.2 (22) and the virulence factor database (23) with an *e*-value cutoff of 10^−10^, similarity ≥ 80%, and coverage ≥ 70%, respectively (8). The abundance of ARGs in each sample was calculated as follows (8):


$$Abundance=\Sigma_{1}^{n}\frac{N_{mapped\;reads}\;\times \;L_{reads}/L_{gene}}{S}\times 10^{6}$$

where *N_mapped reads_* is the number of the reads mapped to ARG-like ORFs, *L_reads_* is the sequence length of the Illumina reads, *L_gene_* is the sequence length of target ARG-like ORFs, *n* is the number of the ARG-like ORFs belonging to the same ARG subtype, and *S* is reads per million.

To explore the putative associations of resistance determinants with MGEs, ACCs were blasted against sequences of integron cassettes (24) and bacterial ICEs (25) with an *e*-value cutoff of 10^−5^, similarity ≥ 85%, and coverage > 60% (26). Plasmid and chromosomal origins of ACCs were predicted by PlasFlow with default parameters (27).

### Metagenomic binning and quality control of metagenome-assembled genomes

The reads of multiple samples were co-assembled using metaSPAdes v.3.13.0, referring to previous studies (28, 29). Metagenome-assembled genomes (MAGs) were recovered using three different tools (MetaBAT2 v.2.12.1, MaxBin v.2.2.6, CONCOCT v.1.0.0) in MetaWRAP (30). The quality of all MAGs was assessed by CheckM v.1.0.12 with lineage-specific markers and filters for low-quality genomes with <50% completeness and <10% contamination (31). RefineM v.0.1.2 was used to improve the completeness of MAGs and decrease contamination (32). The quality score (QS) (defined as completeness − 5 × contamination) was estimated for further MAG quality filtering. Only MAGs with QS ≥50 were used for pairwise comparison and dereplication using dRep v.3.0.0 (33). Finally, 181 dereplicated MAGs were obtained and used for the subsequent analysis.

Taxonomic classification of 181 MAGs was performed using GTDB-Tk v.2.1.0 based on the Genome Taxonomy Database with default parameters (34). High-quality reads of each sample were mapped to all genome sequences (bin fasta) with very sensitive parameters to obtain the mapping bam.files. Based on the mapping bam.files of each sample, we calculated the coverage score for each sample and calculated the average abundance of each MAG. Finally, the relative abundance of each MAG was calculated as the number of reads (based on average coverage) aligned to the MAG normalized by the total number of reads in the sample. ARGs carried by MAGs were identified using BLASTX against the SARG database v.2.2.

### Community-level horizontal gene transfers among different locations

All generated non-redundant MAGs were inferred as horizontal gene transfers (HGTs) at 10 sites using MetaCHIP v.1.10.12 (35). The input files include a folder that holds the sequence file of all query genomes, as well as a text file that holds the taxonomic classification of all input genomes that were classified using GTDB-Tk v.2.1.0. PI and BM modules were used to detect HGTs among customized groups.

### Statistical analysis

Normally distributed data were analyzed using the Student’s *t*-test, while non-normally distributed data were analyzed using the non-parametric Kruskal-Wallis rank sum test and the two-sided Wilcoxon rank sum test in RStudio. A *P* value of <0.05 was considered statistically significant.

### Sequencing data

A total of 987,649,795 reads were obtained from 42 samples, with an average of 23,515,471 for each sample (Table S1). After removing the low-quality reads, 803,160,428 high-quality reads were used for the subsequent analyses. All high-quality reads were assembled into 1,230,803 contigs (>1,000 bp). Prodigal predicted 4,083,859 ORFs (Table S1).

### Relative abundance and diversity of the microbial community in hospital sewage

**FIG 1:**
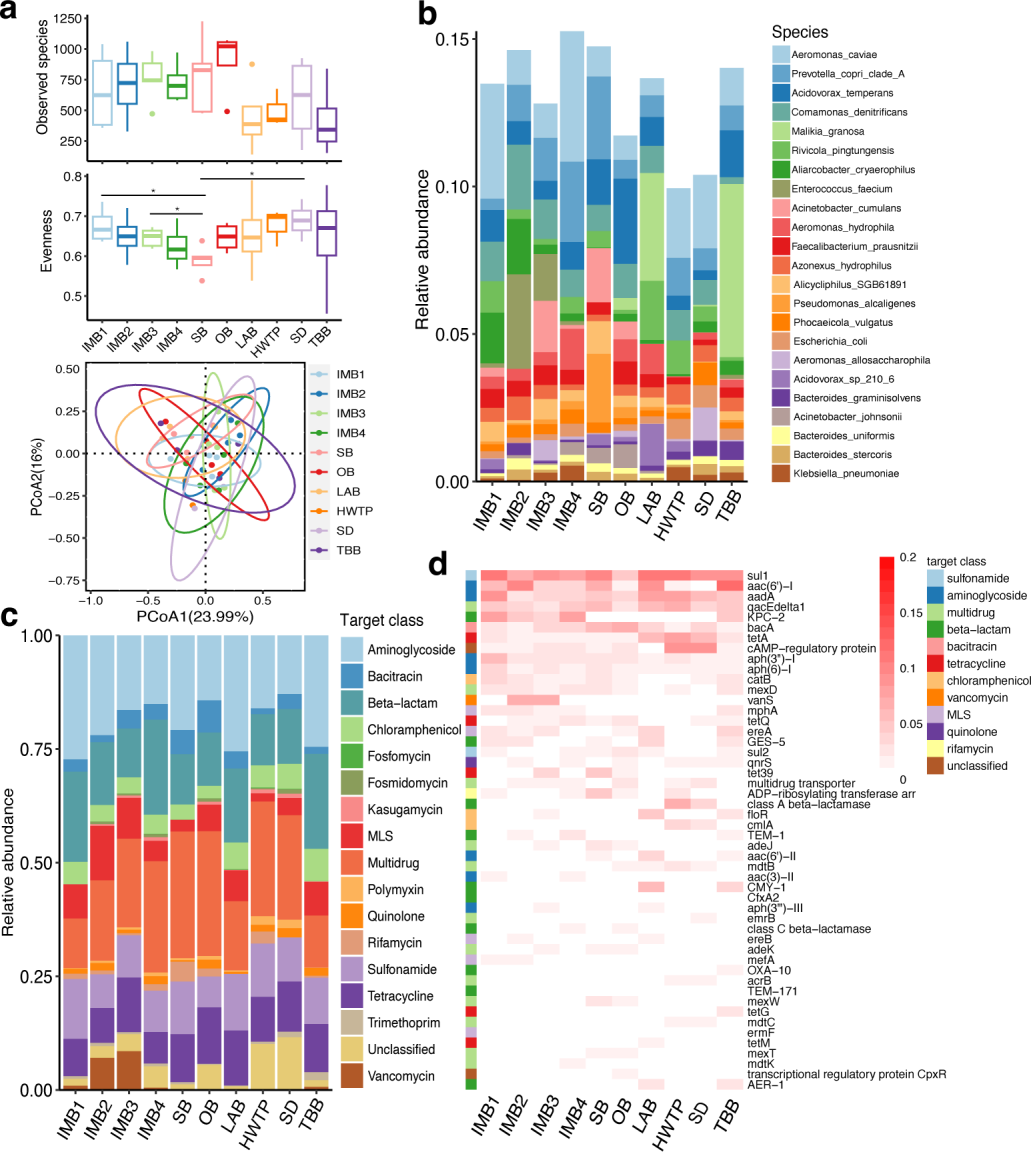
The profile of the microbiome community and ARGs in the sewage of a specialized hospital in Shanghai. (**a**) Alpha and beta diversity of the microbiome community in the sewage of each site. (**b**) Relative abundance of bacterial species in the sewage of each site. (**c**) Relative abundance of ARGs in the sewage of each site. (**d**) The relative abundance of the top 50 ARGs at each site. MLS, macrolide-lincosamide-streptogramin.

Alpha diversity analyses showed that observed species of bacteria at LAB, HWTP, and TBB were less than other sites, and the evenness of bacteria at IMB1, IMB3, and SD was significantly higher than that at SB (*P* < 0.05) (Fig. 1a). Principal coordinate analyses (PCOA) showed the microbial community of each site was similar. Alpha and beta diversity did not significantly change over time (Fig. S2), suggesting that the season has less influence on the microbiome in sewage. We identified 18 bacterial phyla and 1,284 bacterial species. Among them, *Proteobacteria* (53.1%), *Bacteroidetes* (16.7%), and *Firmicutes* (14.5%) were dominated in sewage, followed by *Actinobacteria* (2.6%). At the species level, the relative abundance of *Aeromonas caviae* was highest in each site, followed by *Prevotella copri* clade A and *Acidovorax temperans* (Fig. 1b). *Enterococcus faecium*, *E. coli*, *Klebsiella pneumoniae*, *Pseudomonas aeruginosa*, *Enterobacter cloacae*, and *Aeromonas caviae* were also detected in hospital sewage (Fig. 1b), which are pathogenic bacteria frequently isolated from clinical samples.

### Diversity of ARGs and MGEs in hospital sewage

Overall, we identified 4,530 ARG-like ORFs from 1,230,803 contigs, accounting for 0.4% (Table S1). Subsequently, 252 ARG subtypes identified in hospital sewage belong to 17 antibiotic classes. Among them, multi-drug resistance genes had the highest relative abundance (20.3%), followed by ARGs against aminoglycoside, beta-lactam, sulfonamide, and tetracycline (9.7%-19.1%) (Fig. 1c). Among ARG subtypes, sul1 had the highest abundance (8.5%) (Fig. 1d). In addition, aac(6′)-I had the highest abundance (11.9%) at TBB, and KPC-2 had higher abundance at IMB1, IMB2, IMB3, IMB4, and TBB (3.4%-7.5%) than at the other five sites (0-0.6%) (Fig. 1d).

**FIG 2:**
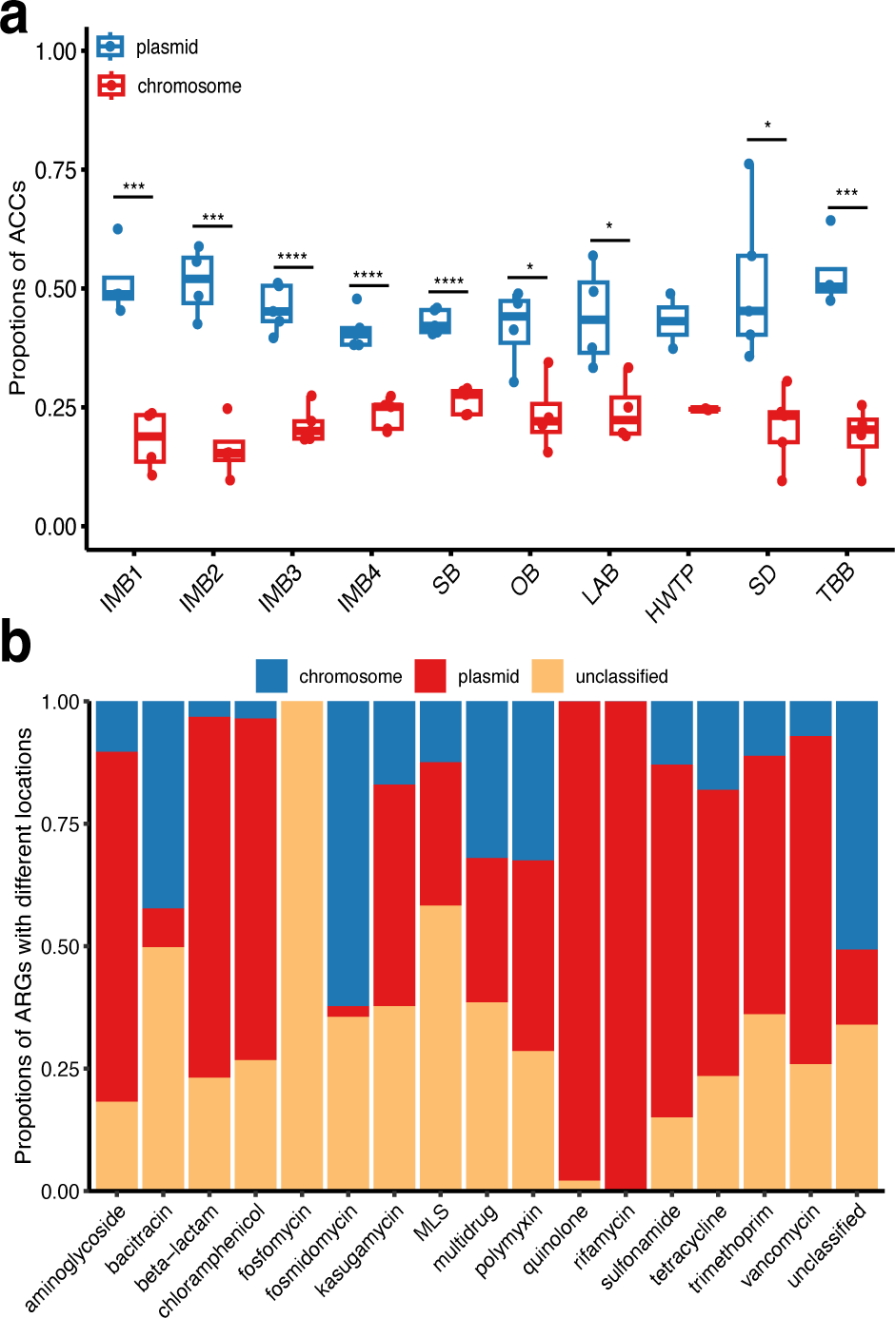
The characteristics of ARG-carrying MGEs. (**a**) Proportions of plasmid- and chromosome-associated ACCs. Comparisons were performed using the Student’s *t*-test. *P < 0.05, ***P < 0.001, ****P < 0.0001. (**b**) The ARG characteristics of plasmid-associated, chromosome-associated, and unclassified ACCs.

Of the ARG-carrying contigs, 47.3%-62.6% were identified as MGEs, and there was no significant difference in the number of MGEs among different sites (Fig. S3). Furthermore, 49.8% and 20.0% of ACCs were classified as plasmid and chromosomal sequences, respectively. The proportion of plasmid-related ACCs was significantly higher than that of chromosome-related ACCs (*P* < 0.05) at all sites, except HWTP (Fig. 2a). Plasmid was the most frequently identified MGE, followed by ICEs (Fig. S4). The resistance genes against aminoglycoside (75.2%), beta-lactam (68.7%), chloramphenicol (76.8%), kasugamycin (38.2%), macrolide-lincosamide-streptogramin (MLS) (47.9%), polymyxin (41.9%), quinolone (90.0%), rifamycin (100%), sulfonamide (65.4%), tetracycline (54.5%), trimethoprim (46.9%), and vancomycin (64.6%) were prevalent in plasmids, while the resistance genes against bacitracin (41.8%), fosmidomycin (70.7%), and multidrug (31.3%) frequently occurred in chromosomes (Fig. 2b).

### Bacterial communities and host-association multiple resistance patterns based on metagenome-assembled genomes

**FIG 3:**
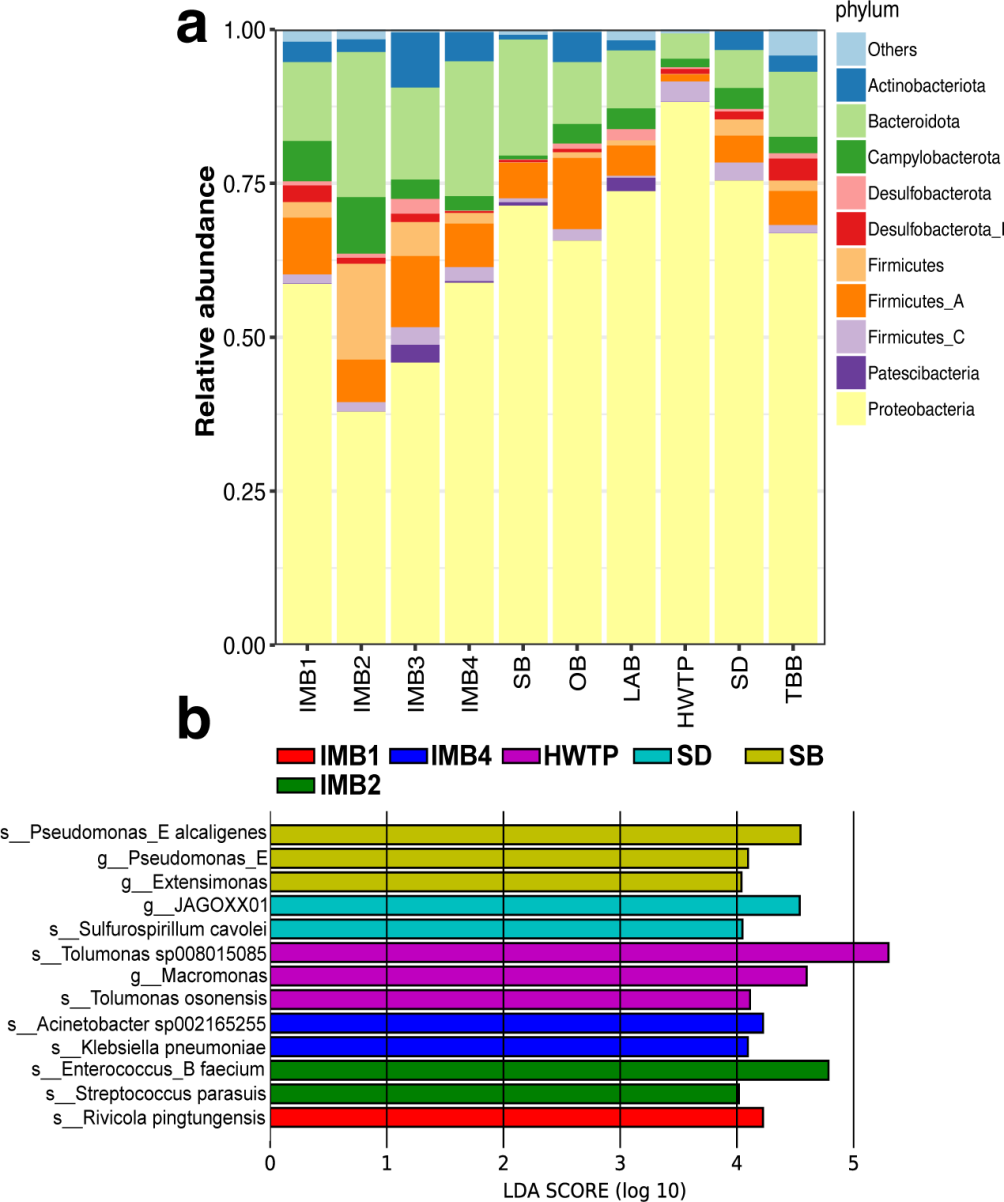
Taxonomic assignment of recovered MAGs. (**a**) Relative abundance of the top 10 phyla. (**b**) Indicator bacteria of different locations identified by the linear discriminant analysis at the species level.

A total of 181 non-redundant MAGs were obtained (Fig. S5; Table S2). Of them, 53 (29.3%) MAGs were evaluated as having ≥90% completeness and ≤5% contamination, 83 (45.9%) had medium quality, and 45 (24.9%) had relatively lower completeness of 50%-70% (Fig. S5). At the phylum level, *Proteobacteria*, *Bacteroidota*, and *Firmicutes_A* were predominant in the communities (Fig. 3a). Lefse analysis showed that IMB2 was associated with the enrichment of *Enterococcus_B faecium* and *Streptococcus parasuis*. *Klebsiella pneumoniae* and *Acinetobacter sp002165255* were significantly enriched in the sewage at IMB4 (Fig. 3b).

**FIG 4:**
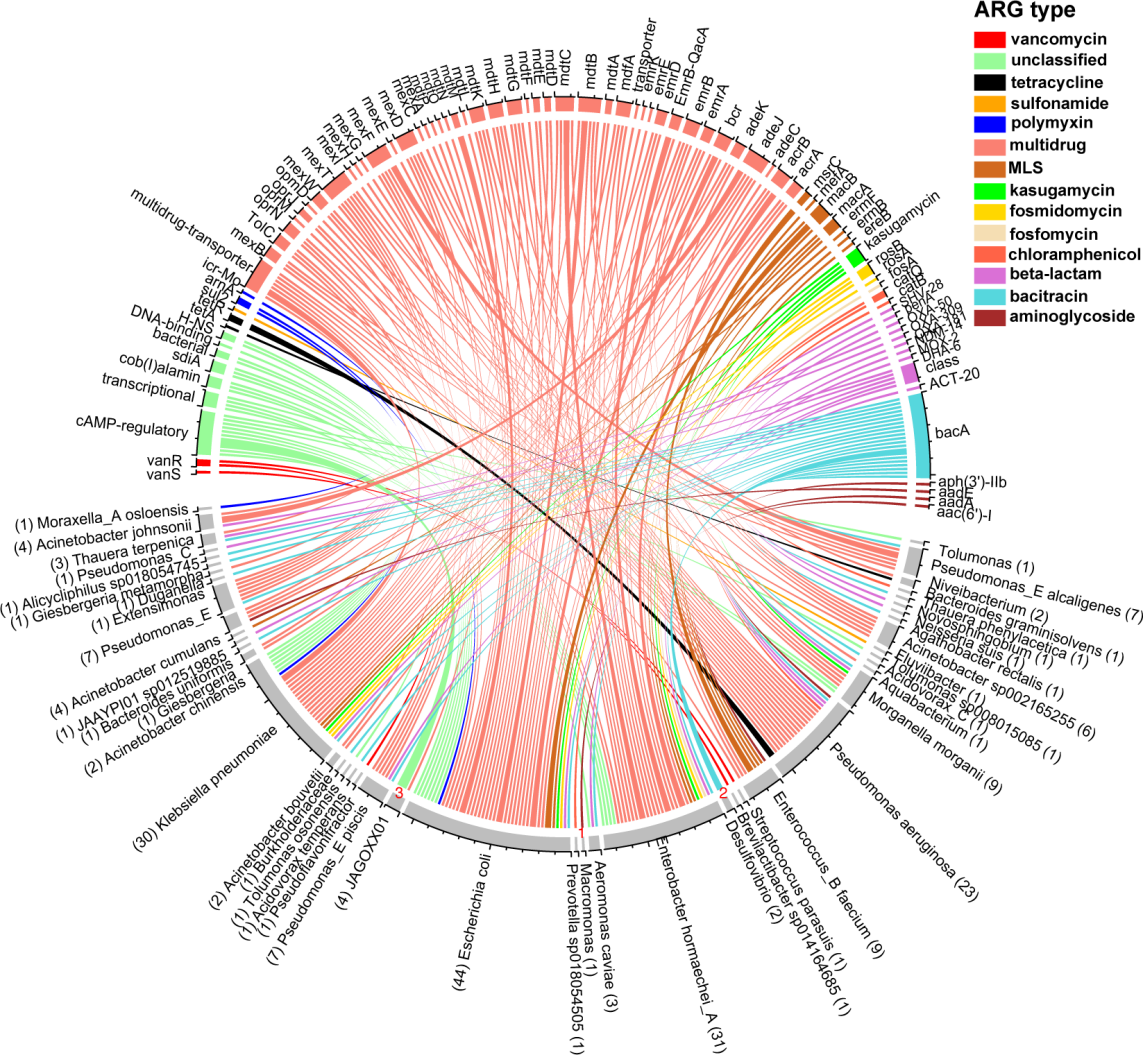
The association between ARGs and hosts. Forty-nine ARG-carrying MAGs and their ARGs were included in the analysis. The data were visualized using RStudio with the R circlize package. In the MAGs sector, the length of the scale on the inner ring represented the total number of ARGs carried by the MAGs. In the ARGs sector, the length of the scale on the inner ring represented the number of MAGs that carried certain ARGs. The widths of different degrees of line represent 1, 2, and 3 (corresponding to the number of ARG), respectively.

A total of 49 MAGs were identified as carrying ARGs and were classified into seven phyla. Among them, 36 MAGs belonged to *Proteobacteria* (Table S3). Thirteen ARG types were identified among these MAGs, and ARGs against multidrug, bacitracin, beta-lactam, and MLS were the most frequently identified resistance genes. Furthermore, 19 MAGs were found to carry at least two ARG subtypes, including 15 carrying more than two ARG subtypes (Fig. 4; Table S3). Importantly, we found that most MAGs carrying multiple ARG types were common pathogenic bacteria detected in hospitals, including *Pseudomonas aeruginosa*, *Enterococcus_B faecium*, *Escherichia coli*, and *Klebsiella pneumoniae*. For example, MAG60, which carried six ARG types against tetracycline (tetR), aminoglycoside [aph(3′)-IIb], beta-lactam (OXA-50), multidrug (e.g., mexW, mexC, and oprJ), and chloramphenicol (cat B), was identified as *Pseudomonas aeruginosa* (Fig. 4; Table S3). MAG115, which carried seven ARG types against kasugamycin, multidrug, MLS, bacitracin, polymyxin, beta-lactam, and fosmidomycin, was assigned to *Escherichia coli* (Fig. 4; Table S3).

### Gene flowing

**FIG 5:**
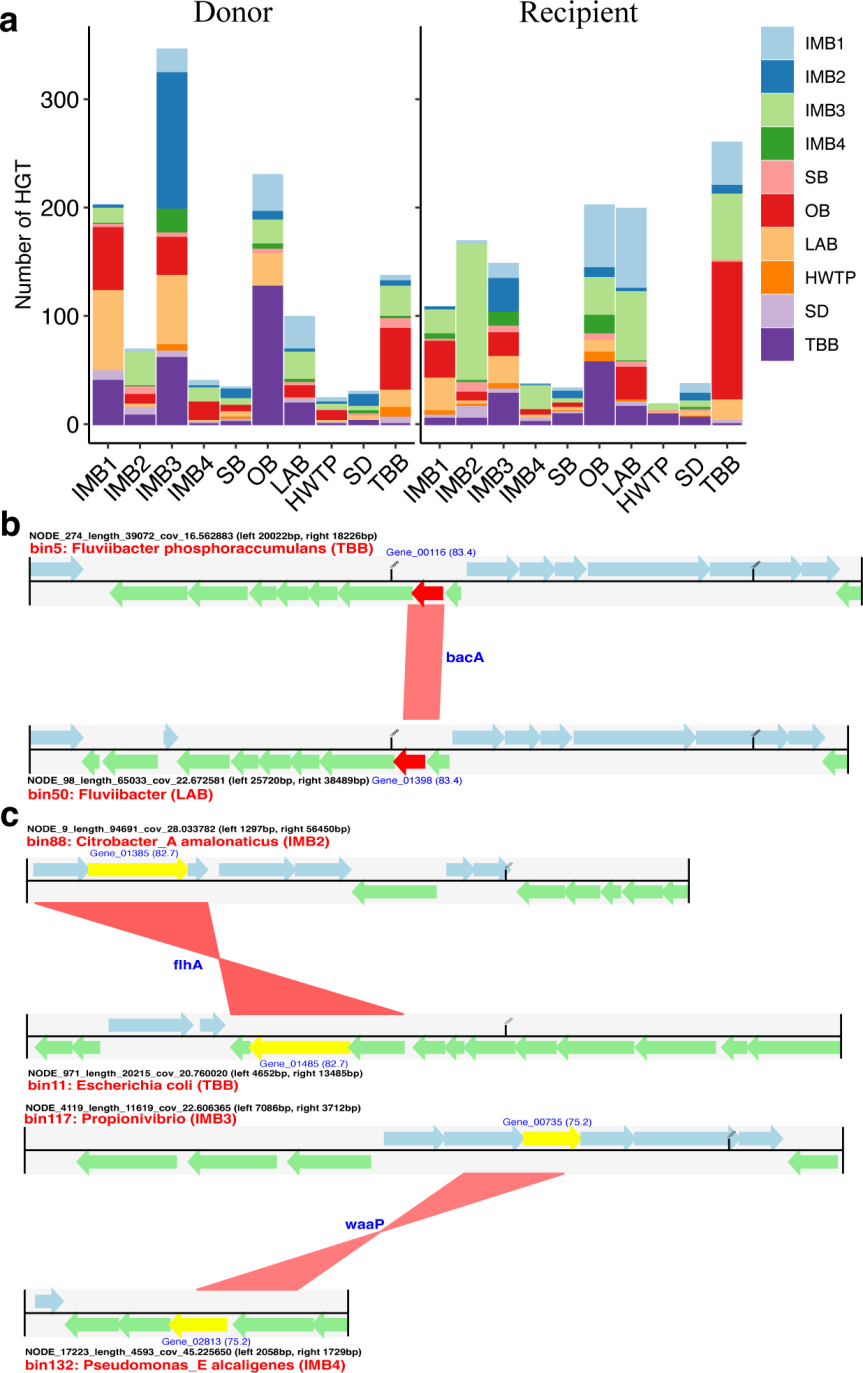
The community-wide HGTs. (**a**) The gene flows among different sites. Metagenome-assembled genomes (MAGs) functioning as donors and recipients were identified, and their corresponding count numbers of HGTs were provided. (**b**) Local genetic structures of the inter-genomic ARG (red) pair. (**c**) Local genetic structures of two representative inter-genomic VF (yellow) pairs. The identities of matching ARGs and gene IDs were annotated. Other ORFs surrounding the inter-genomic ARG or VF are colored in light blue or light green.

The community-level HGTs of MAGs were detected at different sites. Significantly more HGT donors than recipients (70.0% vs 30.0%) were found in sewage at IMB3, suggesting frequent gene flow from IMB3 to other sites. IMB2 (36.4%) appeared to be the primary recipients of IMB3-originated HGTs, followed by LAB (18.5%), TBB (17.6%), OB (10.1%), and IMB1 (6.4%) (Fig. 5a). In addition, it is not surprising that significantly more HGT recipients than donors (71.0% vs 29.0%) were found in sewage at IMB2, and most HGTs (74.6%) were from IMB3. Only one inter-genomic resistance gene against bacitracin (bacA) appeared to be disseminated between *Fluviibacter phosphoraccumulans* at TBB and *Fluviibacter* at LAB (Fig. 5b). Eleven VFs were identified to be involved in HGT events. Two representative HGTs of intra-genomic virulence genes were flhA between *Citrobacter_A amalonaticus* at IMB2 and *Eschrtichia coil* at TBB and waaP between *Propionivibrio* at IMB3 and *Pseudomonas_E alcaligenes* at IMB4 (Fig. 5c).

### Discussion

The presence of ARB and ARGs in hospital environments increases the risk of hospital-acquired infections and brings a huge challenge for clinical management and treatment of infectious diseases (7). Long-term survival of ARB and other pathogenic bacteria in hospital sewage even after sewage treatment processes can cause serious pollution of other water bodies, which poses a huge threat to public health (11). In this study, we investigated the microbiome in the sewage of a specialized hospital (SPHCC) for infectious diseases in Shanghai and revealed the profiles of ARGs and MGEs in hospital sewage.

The ARG profile in the sewage of SPHCC was characterized by the enrichment of aminoglycoside, beta-lactam, sulfonamide, and tetracycline ARGs, with the highest abundance of multidrug ARGs, which is slightly different from the profile in sewage from a hospital in Shantou, a city in South China, where sulfonamide and tetracycline ARGs were enriched and aminoglycoside-resistant genes had the highest abundance (36). The difference might be ascribed to the different disease types covered by the hospitals in Shanghai and Shantou. The hospital in Shantou is an urban general hospital that receives patients with various diseases, whereas SPHCC is specialized for major infectious diseases caused by HIV-1, HBV, and TB, etc. Patients infected with HIV-1, HBV, or TB are often susceptible to acquiring infections by fungi (e.g., *Pneumocystis pneumonia* and *Penicillium marneffei*) and pathogenic bacteria (e.g., *Mycobacterium tuberculosis*, *Klebsiella pneumoniae*, and *Staphylococcus aureus*) due to impaired immune response or immunosuppression (37, 38). Sulfonamides inhibit dihydrofolic acid biosynthesis in Gram-negative and Gram-positive bacteria (39, 40). Sulfamethoxazole belongs to sulfonamide and is commonly used for routine treatment and prophylaxis against infections with *Pneumocystis pneumonia* (41, 42). Sul1 is assigned to resistant sulfonamides and is widely prevalent in environments including rivers, soils, farms, and even effluents of wastewater treatment plants (39). Two previous studies investigated the prevalence and composition of ARGs in the Huangpu River (drinking water source) and the activated sludge of local wastewater treatment plants (WWTP) in Shanghai (43, 44). The detection frequencies of 11 ARGs (sul1, sul2, tetA, tetB, tetC, tetG, tetM, tetO, tetW, tetX, and TEM) in the Huangpu River ranged from 42.86% to 100%. The dominant ARGs in activated sludge encoded resistance to sulfonamide, penicillin, tetracycline, erythromycin, and fluoroquinolone (40.9%-55.4%). Both studies reported the highest abundance of sulfonamide ARGs. Therefore, it is not surprising that sul1 also had the highest abundance in hospital sewage.

We also detected two common aminoglycoside resistance genes, aac(6′)-I and aadA, in the sewage. The aminoglycoside drug streptomycin is a suitable alternative for treating *Mycobacterium tuberculosis* when patients develop resistance to isoniazid or have tolerance to initial treatment (6). A high abundance of aac(6′)-I in the sewage was observed at TBB. Another ARG, KPC-2, confers resistance to beta-lactam and was widely distributed in the sewage at IMBs and TBB. KPC-2 had the highest abundance at IMB4, where the gastroenterology department is located. Furthermore, *Klebsiella pneumoniae* of the Enterobacteriaceae family was found to be enriched in the sewage at IMB4. The wide presence of KPC-2 in hospital sewage is consistent with the increasing trend of β-lactamases in carbapenem-resistant Enterobacteriaceae in China, especially KPC-2 (class A carbapenemase) (45–47).

A high proportion of ARG-carrying contigs identified in sewage were found to carry MGEs, and plasmid-related MGEs accounted for about half, indicating that plasmids played an important role in the transmission of antibiotic resistance. Moreover, plasmid-originated MGEs were associated with multiple ARG types, including aminoglycoside, beta-lactam, chloramphenicol, quinolone, rifamycin, sulfonamide, and tetracycline, which is consistent with previous observations in wastewater treatment plants (8, 48).

It is crucial to understand the correlation between ARB and ARGs in hospital sewage. A previous study reported that *E. coli* can carry multiple ARGs, including AAC(6′)-30/AAC(6′)Ib′, acrD, acrF, baeR, CRP, cmlA5, emrA, marA, patA, pmrF, floR, and/or pp.flo, showing multidrug resistance characteristics (36, 49). The multidrug resistance phenomenon was also observed in bacteria (especially potential pathogenic bacteria) in hospital sewage (36). For example, *Klebsiella pneumoniae* had seven ARGs against bacitracin (bacA), multidrug (e.g., mdfA, mdtH, acrA), MLS (macA, macB), beta-lactam (SHV-28), kasugamycin, fosmidomycin (rosA, rosB), and polymyxin (amA). As a common opportunistic pathogen in hospital environments, *Klebsiella pneumoniae* can cause various diseases such as urinary tract infection, intestinal infection, and pneumonia (50). The widespread use of broad-spectrum antibiotics such as β-lactam and aminoglycosides in the treatment of *Klebsiella pneumoniae* infection inevitably resulted in the generation of carbapenem and other drug resistance (46). Although the number of ARGs from the same bacteria genome might be overestimated due to the co-assembly approach from multiple samples, the identification of multiple ARGs in *Klebsiella pneumoniae* in hospital sewage highlights a growing concern about the clinical management and treatment of multidrug-resistant *Klebsiella pneumoniae*. Furthermore, these results suggested a complex link between ARGs and their hosts’ co-evolution in hospital sewage.

On the other hand, the same ARGs were found in diverse bacteria, suggesting frequent HGTs. Frequent HGTs were more likely observed from IMB3 to IMB2. The main reason might be that IMB3 and IMB2 share the same building (Fig. S1a). IMB3 is responsible for enrolling and treating HIV-1-infected inpatients, and IMB2 is for patients infected with other pathogens rather than HIV-1/HBV/HCV/SARS-CoV-2/TB. Frequent gene flow from IMB3 to IMB2 might imply a greater burden of opportunistic infections in AIDS patients.

This study has three major limitations. First, traditional culture methods were not used to isolate the bacteria from the sewage and further evaluate their antibiotic resistance. The use of a co-assembly approach from multiple samples to recover high-quality genomes might result in an overestimation in the number of multiple ARGs in a bacteria’s genome. Furthermore, to better understand the emergence and transmission of ARB and ARGs, comparative genomic analyses of ARB from both patients and the sewage should be performed. Second, this study is a single-center study conducted in a specialized hospital for infectious diseases. The results only partially reflect the prevalence of ARB and ARGs in hospitals in Shanghai. In addition, the lack of relative data in the sewage of other hospitals and communities limited our ability to compare the ARBs and ARGs among different sites and environments in Shanghai, which can be conducted in future research. Third, the sample size was relatively small in this study since SPHCC is a designated hospital for COVID-19 in Shanghai, and the sewage sampling had been forced to stop since March 2022 under the COVID-19-related pandemic policy. Therefore, we were unable to complete long-term monitoring of ARB and ARGs in the sewage of this hospital, even though no obvious changes in the microbiome were observed in the sewage during almost 1 year.

### Conclusions

We present the characterization profiles of ARGs and MGEs in hospital environments in Shanghai. A total of 252 ARG subtypes across 17 antibiotic classes were identified in hospital sewage. ARG-carrying contigs were often associated with MGEs, especially plasmids, and multidrug resistance was frequently observed in bacteria in hospital sewage. HGTs frequently occurred at the sewage junction of the same building. This study highlights the importance of monitoring ARB and ARGs in hospital environments and suggests a growing concern for the clinical management and treatment of ARB, especially multidrug-resistant bacteria.

## Data Availability

The raw data were deposited in the Sequence Read Archive (SRA) repository under the accession number PRJNA909358.

## SUPPLEMENTAL MATERIAL

Supplemental figures spectrum.03900-23-s0001.docx Fig. S1 to S5.

Supplemental tables spectrum.03900-23-s0002.xlsx Table S1 to S3.

ASM does not own the copyrights to Supplemental Material that may be linked to, or accessed through, an article. The authors have granted ASM a non-exclusive, world-wide license to publish the Supplemental Material files. Please contact the corresponding author directly for reuse.
