# Supplementary material for: The microbiome, resistome, and their co-evolution in sewage at a hospital for infectious diseases in Shanghai, China
Source: Microbiol Spectr. 2023 Dec 22;12(2):e03900-23. doi: 10.1128/spectrum.03900-23 (PMC10846037; doi:10.1128/spectrum.03900-23)
Supplement: Supplemental figures — Fig. S1 to S5. [file spectrum.03900-23-s0001.docx]

Supplementary Material

**The resistome, virulome, and microbiome in sewage at a hospital for infectious diseases in Shanghai, China**

Yingying Ma^1, †^, Nannan Wu^1, †^, Tao Zhang^2^, Yanpeng Li^1^, Le Cao^1^, Peng Zhang^1^, Zhigang Zhang^2, *^, Tongyu Zhu^3, *^ Chiyu Zhang^1, *^

^1^Shanghai Public Health Clinical Center, Fudan University, Shanghai 201508, China

^2^State Key Laboratory for Conservation and Utilization of Bio-Resources in Yunnan, School of Life Sciences, Yunnan University, Kunming, Yunnan 650091, China

^3^Shanghai Key Laboratory of Organ Transplantation, Zhongshan Hospital, Fudan University, Shanghai 200032, China

^†^ These authors contributed equally to this work.

*Correspondence: Chiyu Zhang, Shanghai Public Health Clinical Center, Fudan University, Shanghai 201508, China. Email: [chiyu_zhang1999@163.com](mailto:chiyu_zhang1999@163.com)

Or Tongyu Zhu, Shanghai Key Laboratory of Organ Transplantation, Zhongshan Hospital, Fudan University, No. 180 Fenglin Road, Shanghai 200032, China. Email: [tyzhu@fudan.edu.cn](mailto:tyzhu@fudan.edu.cn)

Or Zhigang Zhang, State Key Laboratory for Conservation and Utilization of Bio-Resources in Yunnan, School of Life Sciences, Yunnan University, Kunming, Yunnan 650091, China. Email: [zhangzhigang@ynu.edu.cn](mailto:zhangzhigang@ynu.edu.cn)


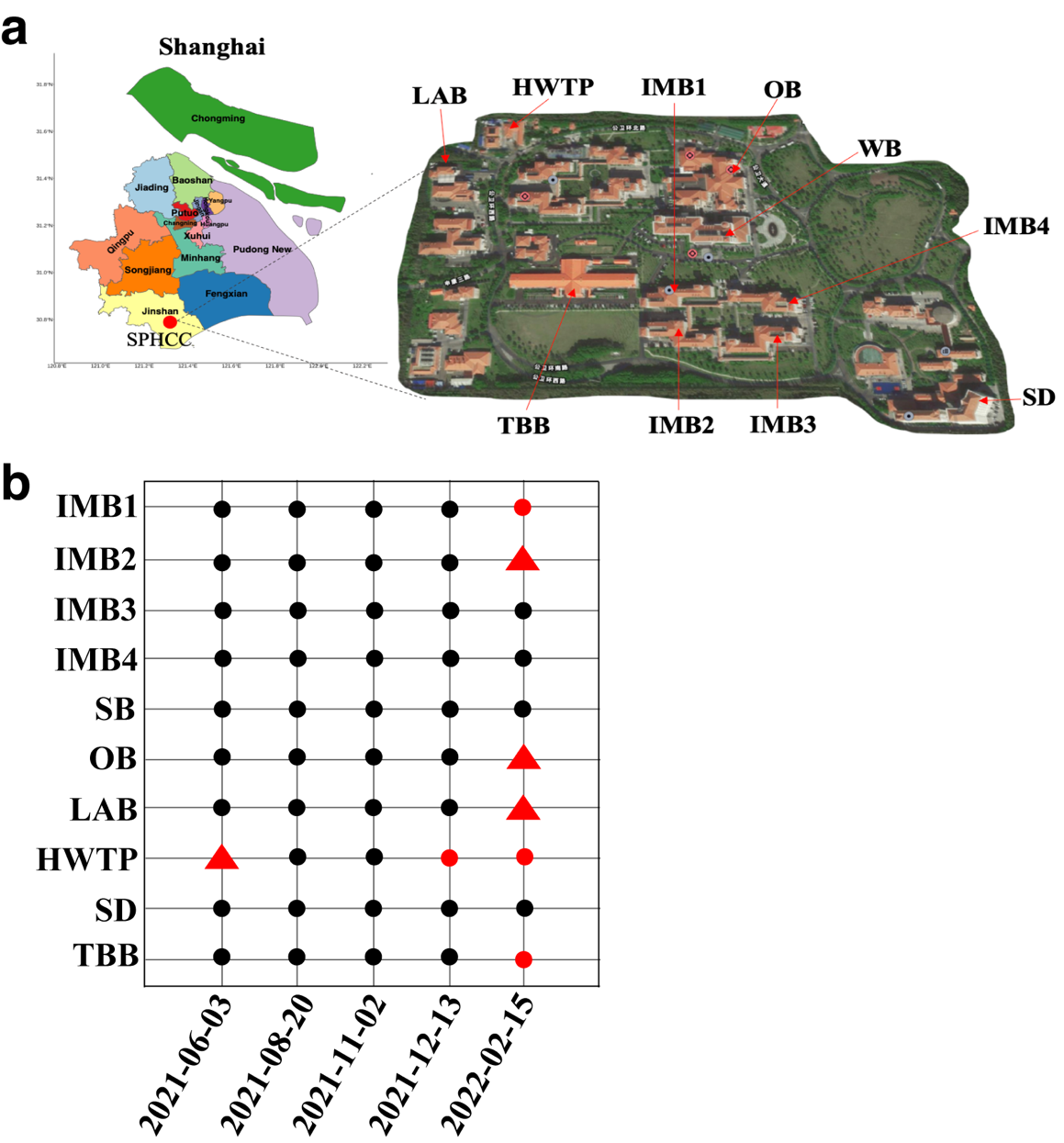


**Figure S1. The map of hospital and distribution of the sampling.** (a) Location of the hospital and the map of ten sampling sites. (b) The sampling sites and time form June 2021 to February 2022. Black dots marked the samples included in this study; red dots marked the samples that weren’t collected; red triangle represent for sequencing failure because of low-quality DNA. SPHCC: Shanghai Public Health Clinical Center; IMB1: Internal Medicine building 1 (Liver Disease); IMB2: Internal Medicine building 2 (including Department of Infectious Diseases, Neurology Department, and Respiratory Medicine); IMB3: Internal Medicine building 3 (AIDs center ); IMB4: Internal Medicine building 4 (Department of Gastroenterology ); SB: Surgery building; OB: Outpatient building; LAB: Laboratory Animal building; HWTP: Hospital Wastewater Treatment Plant; SD: Staff dormitory; TBB: Tuberculosis building.

**
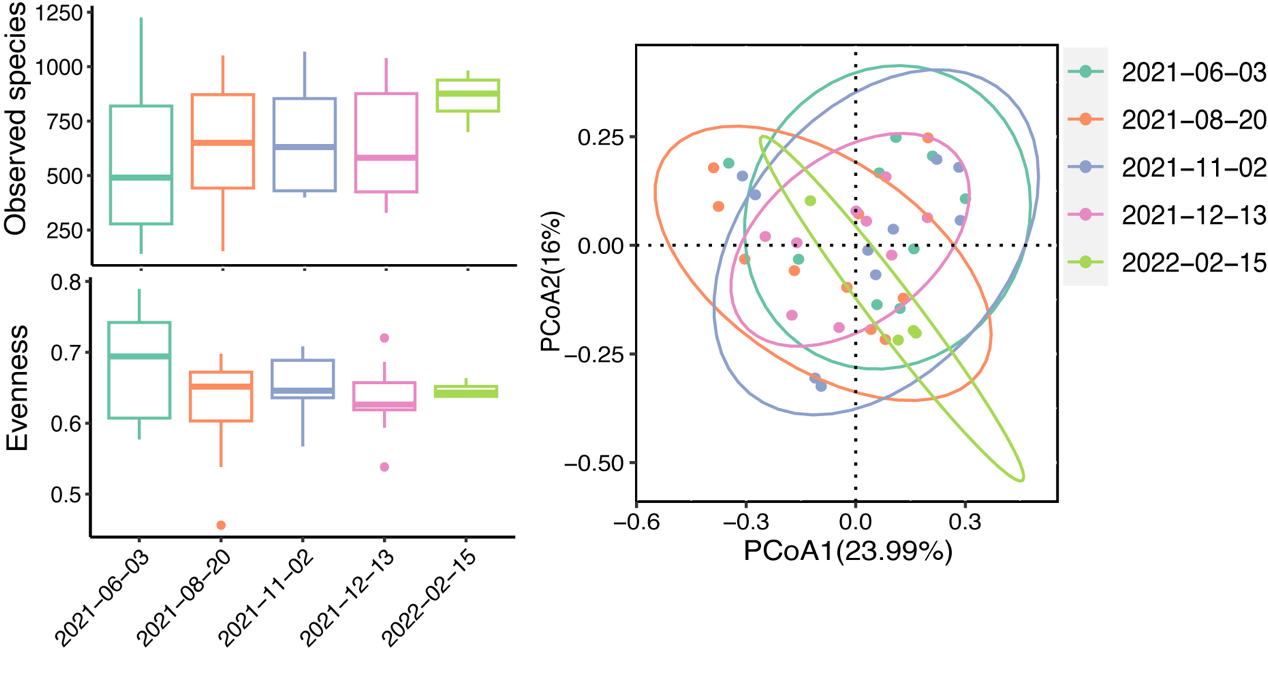
**

**Figure S2. Alpha and beta diversity of microbiome community in hospital sewage over time.**

**Figure S3. Log10 of the counting of contigs, ARGs-carrying contigs and contigs with ARGs that aligned with any MGE.** One contig can be related to more than one MGEs. MGE: mobile genetic element. ICE: integrative and conjugative element.

**Figure S4. Log10 of the counting of contigs, ARGs-carrying contigs and contigs with ARGs that aligned with any MGE.**

**Figure S5. Estimated completeness of 181 identified MAGs.** Genome quality was defined as completeness − 5×contamination, and only the genomes with quality of ≥ 50% were retained. Near-complete genomes (completeness ≥ 90%; contamination ≤ 5%) are shown in red, medium-quality genomes (completeness ≥ 70%; contamination ≤ 10%) in blue, and partial genomes (completeness ≥ 50%; contamination ≤ 4%) in black.
